# Diabetic and ER-stressed pancreatic islet **β** cells are in need of some JNK removal

**DOI:** 10.1172/JCI209808

**Published:** 2026-08-03

**Authors:** Jonathan M. Palozzi, Pere Puigserver

**Affiliations:** 1 Department of Cancer Biology, Dana-Farber Cancer Institute, Boston, Massachusetts, USA.; 2 Department of Cell Biology, Harvard Medical School, Boston, Massachusetts, USA.

## Abstract

Pancreatic β cells regulate glucose homeostasis through insulin secretion, but nutrient overload and genetic defects can trigger ER stress and apoptosis, contributing to type 2 diabetes. Within β cells, the kinases PERK, IRE1α, and ATF6 initiate the unfolded protein response (UPR) as a result of ER stress, a process that is constitutively suppressed under nonstress conditions by GRP78 binding to these proteins. To gain insight into the mechanisms of β cell death upon dysregulated ER stress, Sharma et al. used β cell–specific GRP78 knockout models, revealing that hyperactivation of the UPR promoted β cell death primarily through the IRE1α/JNK/p53 signaling pathway. Pharmacological inhibition of JNK improved β cell survival, increased insulin levels, and lowered blood glucose in multiple diabetic mouse models. These findings highlight JNK signaling as a promising therapeutic target for preserving β cell function.

## Glucose homeostasis, insulin, and diabetes

Precise control of blood glucose is essential for organismal fitness, and its dysregulation leads to a host of chronic illnesses. The pancreas plays a central role in regulating circulating glucose levels via release of insulin (1). β cells within pancreatic islets produce and secrete insulin in response to elevated blood glucose and lipids, and impaired or insufficient insulin secretion due to either autoimmune attack or dietary overload and insulin resistance can lead to the development of type 1 (T1D) or type 2 diabetes (T2D), respectively (2, 3). Global lifestyle changes trending toward increased calorie intake and reduced physical activity have considerably increased the incidence of obesity in recent years, leading to a widespread increase in insulin resistance and T2D and presenting a growing public health challenge that requires urgent attention and clinical treatments (4).

## Proteostatic and dietary stress within β cells

Within β cells, insulin is produced and secreted in large quantities through the endoplasmic reticulum (ER) and vesicle trafficking (5). This high burden on ER protein loading and folding, in combination with lipid overload, can frequently induce ER stress pathways in β cells, triggering an unfolded protein response (UPR). Under nonstress conditions, the ER-resident UPR kinases PERK, IRE1α, and ATF6 are constitutively suppressed by binding of GRP78 (encoded by *HSPA5*) (6–8). In the presence of unfolded proteins and accumulation of ER stress, GRP78 is liberated from the UPR kinases. This allows the kinases to initiate a signaling cascade, which upregulates gene expression to attenuate ER loading burden, increase ER size, and restore proteostasis (7). If left unchecked, however, chronic ER stress signaling can lead to cell death, as occurs frequently in β cells during the development of T2D (9, 10).

Genetic or dietary changes that lead to dysregulation of ER stress and increase β cell death therefore represent a major source of T2D etiology and are areas of active research. From a genetic perspective, *Perk^–/–^* and *Xbp1^+/–^* mice were seen to develop hyperglycemia due to dysregulation of protein synthesis and ER stress, leading to a reduction in β cell mass by cell death and decreased insulin secretion (11, 12). These genetic models highlight that precise and dynamic regulation of ER stress in β cells is an essential aspect of cell viability.

From a dietary perspective, increases in nutrients can promote the rapid production of insulin that, in turn, increases ER protein loading and can activate ER stress. Under basal conditions, temporary elevations in glucose and lipids within the blood, such as after a meal, stimulate β cells to produce insulin in a process known as glucose-stimulated insulin secretion (13, 14). However, chronic elevations in blood glucose and lipids, such as what occurs during obesity and T2D, can induce glucolipotoxicity and lead to β cell death (15).

While many of the sources of dysfunction that lead to ER stress activation have been described, the precise mechanisms of β cell death have not yet been well elucidated. Similarly, it remains unknown if mitigation of ER stress is sufficient to deter cell death once ER stress pathways have become hyperactive. Better understanding of these mechanisms may lead to the prevention of β cell attrition and improve insulin secretion, representing a clinical window that could be leveraged to restore glucose homeostasis.

## β cell–specific GRP78 KO reveals mechanisms of cell death

In this issue of *JCI*, Sharma and colleagues used a β cell specific knockout of GRP78 to investigate the mechanisms of β cell death during unresolved ER stress (16). Upon loss of GRP78, hyperactivation of all branches of the UPR is expected. Using this mouse model, the authors observed rapid loss of β cell mass and disruption of the islet architecture that was strongly evident within 4 weeks of birth, coupled with hyperglycemia and lower serum insulin levels. As a reduction in β cell mass could be due to either decreased proliferation or increased cell death, the authors experimentally confirmed that loss of β cell mass was indeed due to increased cell death.

Next, the authors sought to understand the precise mechanism of β cell death during GRP78 loss using ex vivo cultures of dispersed mouse *Grp78^fl/fl^* islet cells treated with adenovirus Cre to stimulate gene editing. The ex vivo system recapitulated the in vivo phenotypes, showing increased ER stress and cell death, primarily through apoptosis. Bulk RNA-seq on the ex vivo GRP78 knockdown β cells also showed similar effects, indicating a transcriptional increase in stress and apoptotic pathway components.

To reduce unregulated ER stress present in GRP78-KO cells, the authors next inhibited canonical UPR pathway components and surveyed changes in cell viability. They found that suppression of IRE1α but not the other UPR kinases, PERK and ATF6, was sufficient to significantly rescue the β cell death ex vivo. Interestingly, they found that loss of the proapoptotic transcription factor CHOP was insufficient to block cell death, counter to what others have previously reported (17). The increase in IRE1α activity corresponded with an increase in phosphorylation of JNK, a well-characterized downstream kinase. Excitingly, inhibition of JNK phosphorylation using JNK-IN-8 was able to reduce cell death, similar to inhibition of IRE1α, providing a basis for further exploring its therapeutic potential. Further detailed motif analysis of RNA-seq data led the authors to find that p53 was downstream of JNK phosphorylation, and that genetic and pharmacological inhibition of p53 was also able to block cell death in both mouse and human GRP78-depletion β cell cultures.

Lastly, the authors explored if these findings were applicable to in vivo models of diabetes. In two different models of β cell death (pancreatic GRP78 KO and Akita mice), dosing of mice with JNK inhibitor after birth was able to reduce β cell death, increase serum insulin, and reduce blood glucose relative to control mice. Moreover, in the case of Akita mice, JNK inhibition led to an subtle increase of β cell mass. This was particularly remarkable because the JNK inhibitor regimen started after birth, during the critical window when β cells were actively dying. These observations suggest that reducing JNK activity alone is sufficient to stave off β cell death, and the latent insulin-producing function of these cells is sufficient to restore some level of serum insulin. In sum, using a powerful combination of both in vivo and ex vivo murine and human models, Sharma et al. begin to shed light on the mysteries of β cell death due to unmitigated ER stress.

While the use of GRP78-based models to study unresolved ER stress is innovative, the relevance of GRP78 to the etiology of T2D remains less clear, as disease-associated genetic variants have not yet been described. Indeed the authors note that increased GRP78 expression is associated with T2D, limiting the application of these findings. In this context, these results highlight the need to resolve the precise timing of ER stress induction, activation of diabetogenic JNK activity, and commitment to cell death within patients with T2D.

## The future of JNK inhibition in diabetes management

**Figure 1:**
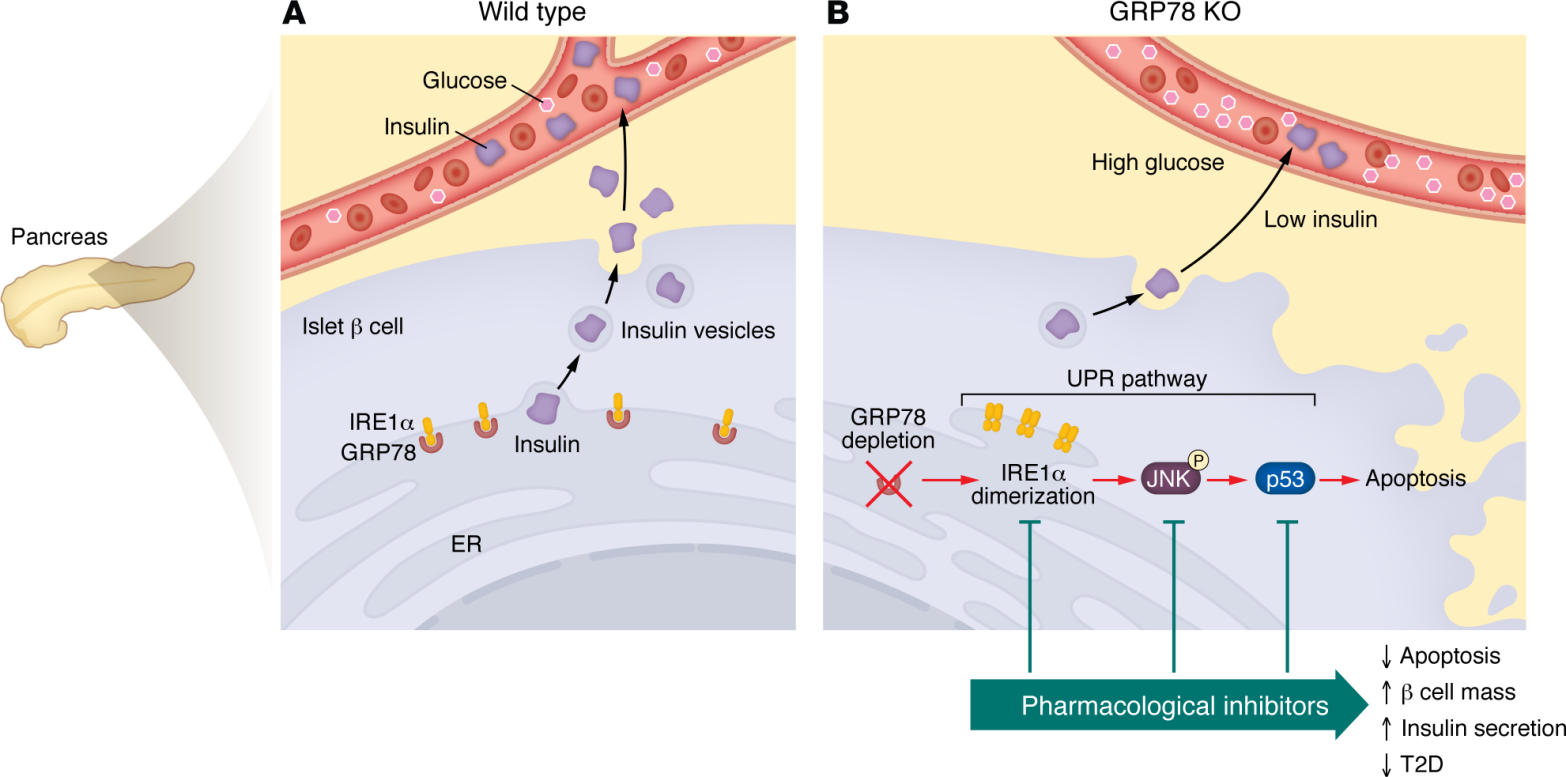
Pharmacological inhibition of the IRE1α-JNK-p53 signaling axis suppresses islet β cell death. Insulin is produced in the ER of islet β cells and is secreted to regulate circulating glucose. Dysregulation of ER stress in islet β cells can be induced by genetic factors or dietary factors, like overnutrition, leading to loss of β cell mass and impairments in insulin secretion and glucose homeostasis. (**A**) Under nonstressed conditions, GRP78 constitutively suppresses dimerization of IRE1α, inhibiting activation of the UPR pathway. (**B**) In Sharma et al.’s study (16), β cell–selective depletion of Grp78 permitted dimerization and activation of the stress kinase IRE1α, which, in turn, phosphorylated JNK, activated p53, and led to apoptosis. Systemic application of pharmacological inhibitors targeting any step in this process reduced β cell death, increased insulin secretion, and had an antidiabetic effect in these mice.

In the absence of underlying defects in insulin production and secretion, hyperactivity of ER stress and subsequent death of β cells can be blunted by treatment with a JNK or p53 inhibitor, allowing for improvements in β cell mass, insulin secretion, and metabolic phenotypes (Figure 1). These findings shed light on unsolved questions, demonstrating that β cell death can be driven exclusively by hyperactivity of stress kinases and that simply reducing activation of apoptotic pathways can appreciably preserve serum insulin levels. While JNK inhibition seems to robustly reduce β cell death, a corresponding correction in β cell mass and normalization of blood glucose was not always apparent, suggesting that a more nuanced relationship may exist. Furthermore, this model of β cell GRP78-KO- and ER stress–driven cell death reflects a T2D system, limiting the applicability of the findings to therapies for T1D. Regardless, these interesting observations allow us to propose some exciting possibilities.

The ability for JNK inhibition to reduce cell death in GRP78-KO dispersed human β cell cultures suggests a degree of conservation of these pathways between mice and humans that may be clinically relevant. Current therapies for improving β cell function and serum insulin levels span a range of approaches, including stem cell therapies to rejuvenate the islet compartment (18), metformin to reduce blood glucose, and GLP1 agonists to stimulate insulin secretion and promote proliferation (19). It is enticing to understand how systemic JNK inhibition may synergize with these standard-of-care treatments, allowing for further improvements in β cell viability and insulin secretion. It will also be interesting to see if there is an age dependence to the glycemia-restorative effects conferred by JNK inhibition, as the latent regenerative capacity of β cells to repopulate the islet niche after death likely decreases with age. Therapeutically, while pan-JNK inhibitors can trigger unpredictable toxicity, the possibility of targeted delivery and use of JNK isoform–specific inhibitors might be a potential strategy to pursue in combination with current antidiabetic treatments (20, 21). Specific delivery of JNK inhibitory therapeutic agents (mRNAs, peptides, or compounds) to β cells through lipid nanoparticles is currently the most promising in its ability to modulate disease progression of T2D (22).

## Conflict of interest

The authors have declared that no conflict of interest exists.

## Funding support

This work is the result of NIH funding, in whole or in part, and is subject to the NIH Public Access Policy. Through acceptance of this federal funding, the NIH has been given a right to make the work publicly available in PubMed Central.

- NIH grants R01 CA181217-10, R01 DK136640-02, R01 AG086369-02, and R01DK142741.

## Version 1 08/03/2026

Electronic publication
